# The nature of the erythrocyte coating substance in tumour-bearing rats.

**DOI:** 10.1038/bjc.1968.100

**Published:** 1968-12

**Authors:** H. M. Anthony

## Abstract

**Images:**


### THE NATURE OF THE ERYTHROCYTE COATING SUBSTANCE IN TUMOUR-BEARING RATS

HONOR M. ANTHONY

*From the Department of Experimental Pathology and Cancer Research, School of Medicine, Leeds, 2*

Received for publication August 8, 1968

AGGLUTINATION of the erythrocytes of human cancer patients (Green, Wakefield and Littlewood, 1957; Betts *et al.*, 1962; Anthony and Parsons, 1965), of tumour-bearing hamsters (Betts *et al.* 1961) and of tumour-bearing rats (Green, Wakefield and Littlewood, 1957) by antiserum to normal serum globulins has been reported. It is an atypical Coombs' reaction which has been called the erythroagglutination reaction (Betts *et al.*, 1964). Agglutination of cancer patients' erythrocytes was not demonstrable with the commercial antiglobulin antisera (anti-$\gamma$- and anti-non-$\gamma$-) tested, and was lost from laboratory-prepared antiglobulin antiserum as it aged while the anti-D titre was unaffected (Anthony and Parsons, 1965). Some workers have failed to elicit the reaction (Beilby, 1958) while others have demonstrated the reaction only with antisera to the glubulins of cancer patients (Gelhorn, 1960; Sohier, Juranies and Aub, 1957). The suggestion of Beilby (1958) that the reaction demonstrated the adsorption of cold agglutinin can be discounted since it has been demonstrated with erythrocytes prepared immediately in the warm (Anthony and Parsons, 1965).

The transplanted $RD_3$ tumour grows progressively to kill the host in 12–14 days when grown in ascites form in Sheffield Wistar rats, maintained as a closed colony. In about half the rats the erythroagglutination reaction becomes positive 2 or 3 days before death. The nature of the erythrocyte coating substance contributing to this reaction has been investigated.

### MATERIALS AND METHODS

### *Erythrocyte agglutination*

Anti-rat globulin serum was prepared in rabbits by the method of Dunsford and Bowley (1955).

An incomplete anti-erythrocyte antibody was prepared in rats by immunising with crude lipid, prepared by chloroform-methanol extraction of rabbit erythrocyte stroma, suspended in rabbit serum. The most satisfactory antiserum was obtained from rats immunised by subcutaneous injection of 2·4 mg. lipid in 0·5 ml. rabbit serum and 0·5 ml. Freund's complete adjuvant, boosted with 1·2 mg. lipid in serum and adjuvant a week later and bled after a month. This caused no haemolysis or haemagglutination of rabbit erythrocytes after heat inactivation at 56° C. for 30 minutes. Satisfactory sensitisation was obtained by incubation of a 5% suspension of rabbit erythrocytes for 1 hour at 37° C. with serum diluted 1 in 10. These erythrocytes were used as a positive control.

Normal rat and rabbit erythrocytes were included as negative controls.

Erythrocytes were washed three times in isotonic saline before use. The test was performed using doubling dilutions of antiglobulin serum on a white tile which was gently rocked. The result was read after 5 minutes.

*Preparation of the erythrocyte coating substance (ECS)*

Rats showing a positive erythroagglutination reaction (agglutination to a titre of 1 in 4 or more) were exsanguinated from the heart or thoracic cavity. The blood was collected into citrated saline, the erythrocytes separated by gentle centrifugation (200 ***g***), washed 6 times in isotonic saline and resuspended at 20%. Smears of the erythrocyte suspension showed negligible contamination with tumour cells. Elution was carried out by incubating the erythrocyte suspension at 56° C. for 20 minutes with occasional inversion of the tube, followed by immediate centrifugation in buckets containing water preheated to 56° C. The sediment was discarded.

On other occasions, eluate was prepared from erythrocyte stroma. The erythrocytes were lysed by suspension in progressively increasing dilutions of saline in distilled water. Sodium chloride was added to the suspension of stroma to restore isotonicity before centrifugation (6250 ***g*** for 20 minutes). The sedimented stroma was washed twice in large volumes of isotonic saline and resuspended in the volume of saline which would have given a 20% suspension of the original unlysed erythrocytes. After elution as above, the erythrocyte stroma was spun off at 6250 ***g*** and the sediment discarded.

*Preparation of antiserum to ECS*

Rabbits were given 4 weekly intramuscular injections of 1 ml. of ECS in an equal volume of Freund's complete adjuvant, followed by 3 intravenous injections of 0·5 ml. ECS on alternate days, and bled 9 days after the last injection. Individual rabbits were immunised with ECS prepared either from lysed or from whole erythrocytes.

*Absorption of antisera*

Antisera were absorbed with an equal volume of packed normal rat erythrocytes (1 hour at 37° C.) and with either liver, serum or tumour powder at 10 mg./ml. (1 hour at 37° C. and 18 hours at 4° C.). Liver and tumour were passed through progressively finer stainless steel gauzes and washed 6 times in saline and twice in distilled water before lyophilisation; serum was dialysed against distilled water for 18 hours at 4° C. and lyophilised.

*Preparation of tumour coating substance (TCS)*

The ascites tumour $RD_3$ was drained from the peritoneal cavity with a large bore needle and the cells collected by gentle centrifugation. The supernatant was recentrifuged (800 ***g*** for 5 minutes) and the fibrin clot allowed to form. The ascitic fluid (AsF) was drawn off for use in gel diffusion. The cells were resuspended in distilled water to lyse the contaminating erythrocytes and the tumour cells washed 4–6 times in isotonic saline. A 20% suspension was used for elution of tumour coating substance (TCS) as described for erythrocytes.

*Immunodiffusion*

Immunodiffusion (see Ouchterlony, 1962) was carried out using a 1% solution of Ionagar No. 2 (Oxoid) in isotonic saline with or without the addition of 0·2% sodium azide, in petri dishes and on microscope slides. Gels were kept in a moist atmosphere at room temperature and inspected regularly. Large gels were recorded as contact prints on photographic paper at or before the 4th day. Small gels were enlarged directly on to photographic paper, using diffused light, at 20 hours. Gels were subsequently washed in saline followed by distilled water, dried at 37° C. and stained with naphthol black.

The antigens used were ECS, TCS, the serum (AsS) and the ascitic fluid (AsF) of ascites tumour-bearing rats, normal rat serum (NS) and reconstituted pure rat IgG globulin (kindly provided by Immunology Inc. of Chicago). The reactions were compared to those with anti-rat globulin serum.

*Immunoelectrophoresis*

Microelectrophoresis of serum and ascitic fluid was carried out by the method of Wieme (1959) using barbitone buffer pH 8·4, ionic strength 0·1. Satisfactory separation on two slides covered with 0·75 mm. of 0·8% agar in buffer was obtained using a 30 minute run at 150 v, 40–50 mA. After application of the antiserum, gels were developed in a moist atmosphere at room temperature and recorded at 20 hours.

### RESULTS

Agglutination of erythrocytes of tumour-bearing rats was not detected using anti-eluate serum E.

When unabsorbed antiserum (E) to erythrocyte coating substance (ECS) was tested in gel diffusion against ECS, there was a broad band of precipitation which showed a reaction of identity with components of normal serum. This band was less marked when antiserum prepared from lysed erythrocytes was used, or when the ECS used on the plate had been prepared from lysed erythrocytes.

---

### EXPLANATION OF PLATES

FIG. 1 and 2.—Central Well; E, anti-ECS serum.

Peripheral Wells; NS, normal serum; AsS, AsF, serum and ascitic fluid of tumour-bearing rat diluted where indicated; ECS, erthrocyte coating substance; TCS, tumour-coating substance.

FIG. 3 and 4.—Antiserum in side wells; E-S, anti-ECS serum absorbed with freeze dried serum; E-T, anti-ECS serum absorbed with tumour powder; E-L, anti-ECS serum absorbed with liver powder. In each instance the antiserum had also been absorbed with erythrocytes.

Antigens: IgG, reconstituted lyophilised pure rat IgG (kindly provided by Immunology Inc. of Chicago). AsS, AsF, serum and ascitic fluid of tumour-bearing rat; TCS, tumour-coating substance.

FIG. 5 and 6.—Antiserum in side wells; E, anti-ECS serum; ARG, anti-rat globulin serum, diluted where indicated.

Antigens; IgG, reconstituted lyophilised pure rat IgG; AsS, AsF, serum and ascitic fluid of tumour-bearing rat, diluted where indicated; TCS, tumour-coating substance.

FIG. 7.—Immunoelectrophoretic pattern.

Top slide: top well, normal serum; bottom well, serum of tumour-bearing rat. Antiserum, anti-rat globulin serum.

Bottom slide: top well, normal serum; bottom well, tumour-coating substance. Antiserum, anti-ECS serum.

1

2

Anthony.

AsS
E-S
E-L
AsF
E-L
E-S
IgG

3

AsS
E-T
E-L
TCS
E-L
E-T
IgG

4

IgG
E
ARG
TCS
ARG
E
AsS

5

6

7

Anthony.

It was absent when the antiserum had been absorbed with normal erythrocytes and liver powder (E-L).

A fine line, often double, to a slower-moving component of ECS was not affected by such absorption. It showed a reaction of identity with a component of tumour coating substance (TCS) and of the serum (AsS) and the ascitic fluid (AsF) of tumour-bearing rats (Fig. 1) and a reaction of partial identity with reconstituted pure rat IgG globulin. No appreciable deviation of this line on approaching the well containing fresh normal serum was detected, but clear deviation occurred near the well containing serum from a tumour-bearing rat (AsS) diluted 1 in 10 (Fig. 2), indicating that the difference in concentration of this component in the two sera was at least ten-fold. The line was reduced or removed by prior absorption of the antiserum with either serum powder or tumour powder. The presence of this line after absorption with normal erythrocytes and liver powder (E-L) and its reduction after absorption with serum powder (E-S) or tumour powder (E-T) is shown in Fig. 3 and 4. It will be noted that the antiserum absorbed with serum powder (E-S) acts as an additional source of antigen to the antiserum absorbed with liver powder (E-L). This was a regular finding. Fig. 4 also demonstrates the reaction of partial identity with reconstituted IgG globulin.

When the reactions of the antiserum E were compared to that of rabbit anti-rat globulin serum (ARG), there was fusion of a line due to a component capable of reacting with both antisera (Fig. 5 and 6). This showed identity with the component of reconstituted IgG with which the E antiserum reacted. This was shown, from its reaction with ARG antiserum, to be a minor, faster-moving component of reconstituted IgG (Fig. 5).

On immunoelectrophoresis additional lines have been noted in the $\alpha$- and $\beta$-regions when the serum and ascitic fluid of tumour-bearing rats have been allowed to react with anti-rat globulin serum. Weak lines in the $\alpha$ or $\beta$ position have also appeared on development with anti-ECS serum. The top slide of Fig. 7 shows additional lines in the $\alpha$- and $\beta$-regions of ascitic serum (bottom well) not detectable in normal serum (top well) when reacting with anti-rat globulin serum. The lower pattern is that produced by the reaction of TCS with anti-ECS serum; a weak line is detectable in the $\beta$-region. Normal serum (top well) gave rise to no lines against this antiserum.

### CONCLUSIONS

These reactions indicate that the erythrocyte coating substance (ECS) of rats bearing the $RD_3$ ascites tumour and showing a positive erythroagglutination reaction contains an altered globulin. The line of partial identity is with a minor component of reconstituted pure rat IgG not detectable in fresh normal serum. Demonstration of the same antigen in the serum and ascitic fluid of tumour-bearing rats rules out artefactual denaturation in the process of elution as the source of alteration of the globulin. The alteration must therefore have occurred *in vivo* either by aggregation, by partial degradation or by combination with antigen.

For a variety of reasons, the latter seems the most likely explanation. The antigen forms a clear reaction of *partial* identity with the altered component of IgG. Although the line is commonly double, no significant separation of the line has been achieved, and only one line has been detected on immunoelectrophoresis using anti-ECS serum absorbed with liver and erythrocytes. The line was reduced or absent on gel-diffusion using anti-ECS serum absorbed with either tumour or freeze dried serum.

The similarity of reaction patterns and the reactions of complete identity of this antigen in ECS, in tumour-coating substance (TCS) and in the serum and ascitic fluid of tumour-bearing rats indicates that the same antigen is present at at all these sites. It gives no indication of the origin of the complex.

The absence of the complex from normal erythrocytes (as shown by absorption) and the reaction of partial identity of the lines due to the complex detected with anti-eluate and anti-globulin antisera suggest that it is the protein responsible for the atypical agglutination. This could not be confirmed by direct agglutination of the erythrocytes of tumour-bearing rats using anti-ECS serum.

An atypical Coombs' reaction has been reported in the presence of a high reticulocyte count, and has been shown to be dependent on the presence of transferrin on immature erythrocytes (Jandl, 1960). The agglutinated clumps were normally not visible with the naked eye and were almost exclusively composed of reticulocytes. Transferrin is unlikely to be responsible for the erythroagglutination reaction in the tumour-bearing rat for a variety of reasons. The agglutination was always read with the naked eye. The complex detected showed partial identity with a component of freeze-dried serum, was not detected in normal serum but was present in increased amounts in the serum of tumour-bearing rats. By contrast low serum transferrin levels have been noted in cancer patients (Bariety and Gajdos, 1960; Masuya *et al.*, 1963) and in the presence of anaemia due to blood loss or iron deficiency (Masuya *et al.*, 1963). Raised serum transferrin has been reported in rats bearing hepatomata (Clausen *et al.*, 1960), consistent with the presumed hepatic origin of transferrin. In addition, the fact that the complex on the erythrocytes was immunologically identical with and comparable in concentration to the substance coating the tumour cells is not consistent with it being transferrin.

This slowly-diffusing complex with electrophoretic characteristics of a $\beta$-globulin would seem to be distinct from the rapidly diffusing serum glycoprotein described by Darcy (1960, 1965) in the serum of rats with tumours and those with tissue injury or necrosis. If the complex described is an antigen-antibody complex, as it appears to be, it may, in this non-syngeneic situation, be the result of a host response to transplantation antigens rather than a response to the tumour *qua* tumour. This could not be the case in the apparently comparable situation in the human cancer patient.

### SUMMARY

The nature of the erythrocyte coating substance contributing to the positive erythroagglutination reaction in rats bearing the $RD_3$ ascites tumour has been investigated using gel diffusion and immunoelectrophoresis. The results indicate that it is immunologically identical with a component of tumour coating substance, and of the serum and ascitic fluid of tumour-bearing rats, and that it contains an altered globulin.

### REFERENCES

Anthony, H. M. and Parsons, M.—(1965) *Nature, Lond.*, **206**, 275.
Bariety, M. and Gajdos, A.—(1960) *Path. Biol., Paris*, **8**, 709.
Betts, A., Rigby, P. G., Emerson, C. P. and Friedell, G. H.—(1961) *J. Lab. clin. Med.*, **58**, 652.

Betts, A., Rigby, P. G., Friedell, G. H. and Emerson, C. P.—(1962) *Blood*, **19**, 687
Betts, A., Tanguay, R. and Friedell, G. H.—(1964) *Proc. Soc. exp. Biol. Med.*, **116**, 66.
Beilby, J. O. W.—(1958) *Br. med. J.*, ii, 549.
Clausen, J., Rask-Nielsen, R., Christensen, H. E. and Munkner, T.—(1960) *Cancer Res.*, **20**, 178.
Darcy, D. A.—(1960) *Br. J. Cancer*, **14**, 534.—(1965) *Br. J. exp. Path.*, **46**, 155.
Dunsford, I. and Bowley, C. C.—(1955) ' Techniques in Blood Grouping ', Edinburgh (Oliver & Boyd), p. 134.
Gelhorn, A.—(1960) *Proc. int. Soc. Haemat.*, **3**, 694.
Green, H. N., Wakefield, June and Littlewood, G.—(1957) *Br. med. J.*, ii, 779.
Jandl, J. H.—(1960) *J. Lab. clin. Med.*, **65**, 663.
Masuya, T., Kozuru, M., Migita, S., Tominaga, K. and Umeda, T.—(1963) *Kyushu J. med. Sci.*, **14**, 223.
Ouchterlony, O.—(1962) *Prog. Allergy*, **6**, 30.
Sohier, W. D., Juranies, Eleanor and Aub, J. C.—(1957) *Cancer Res.*, **17**, 767.
Wieme, R. J.—(1959) *Clin. chim. Acta*, **4**, 317.


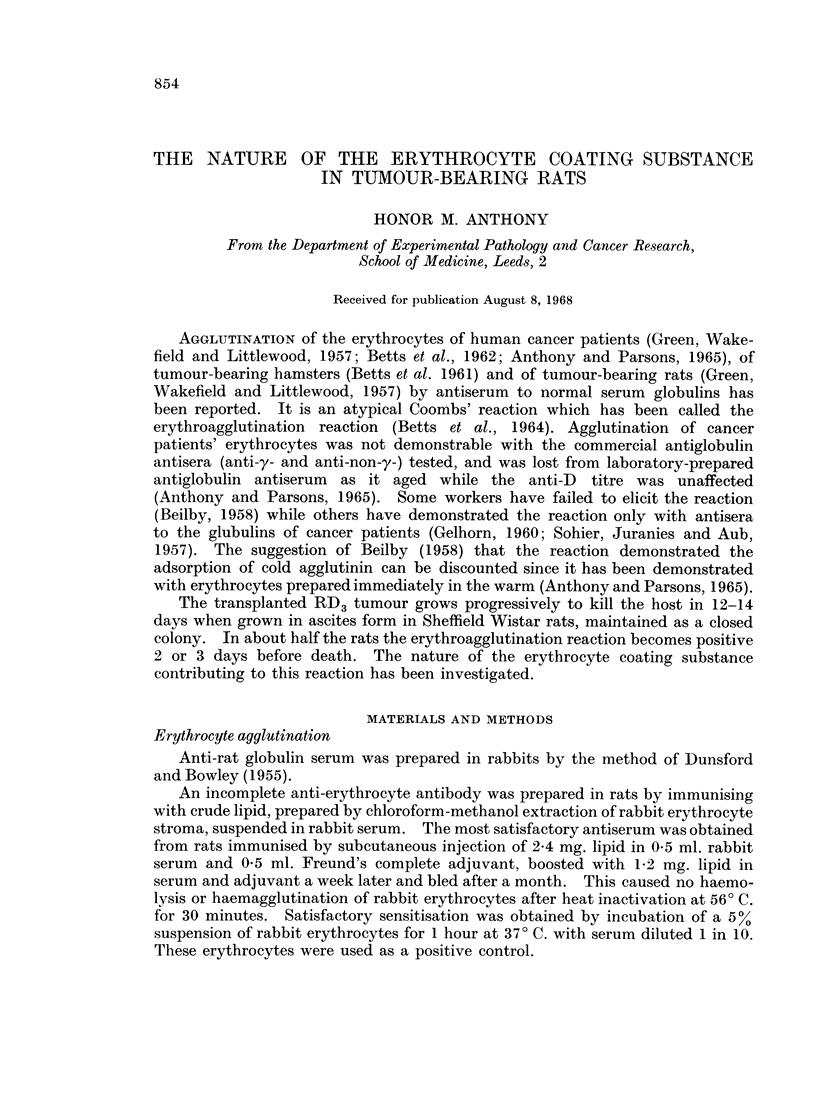

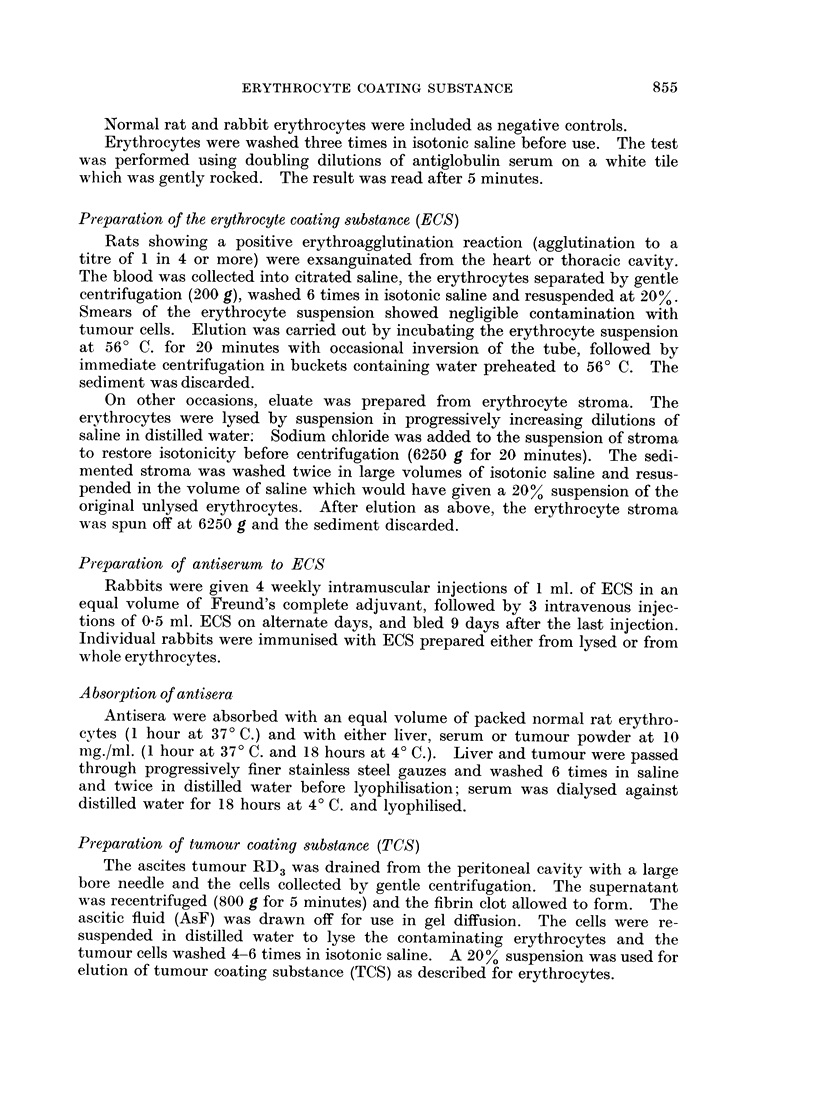

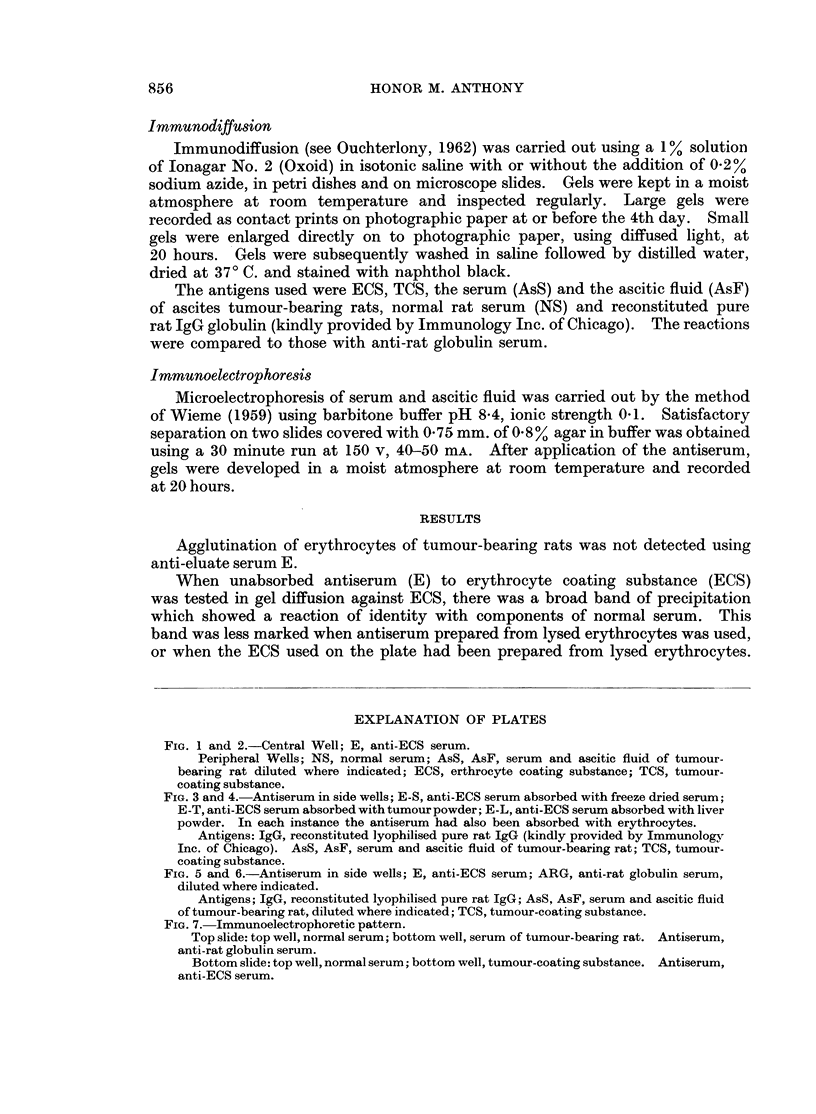

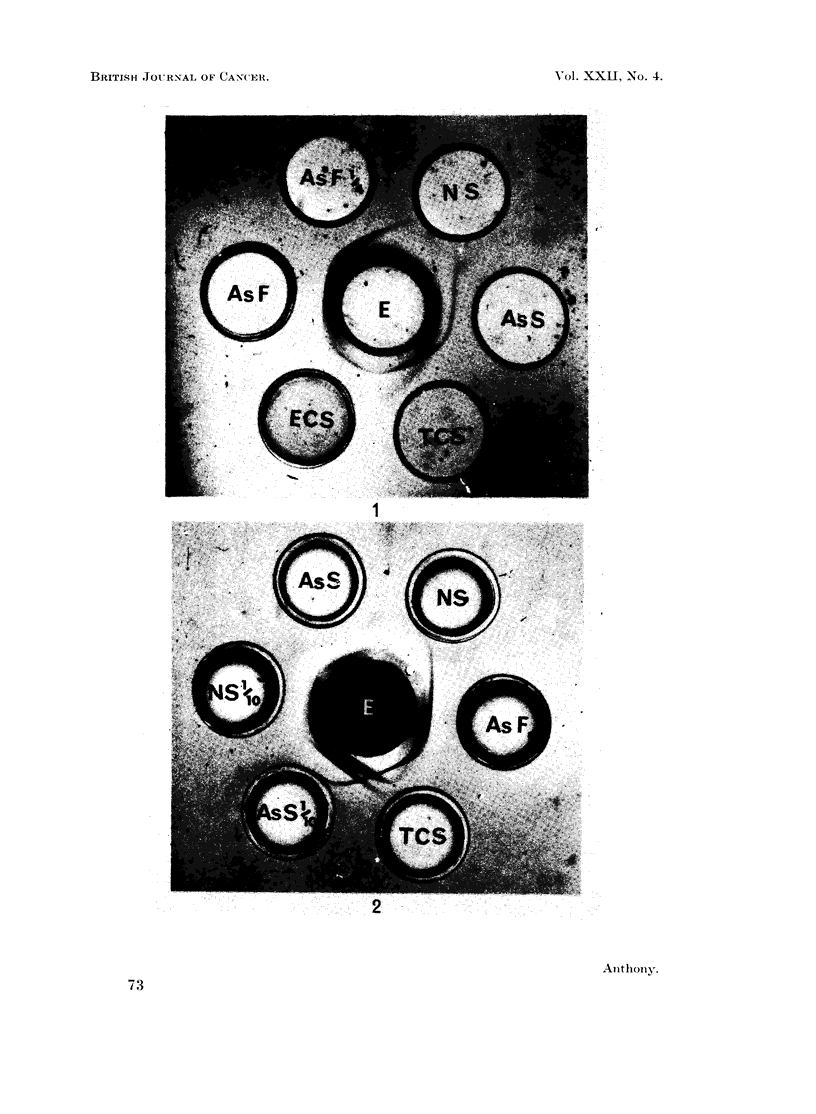

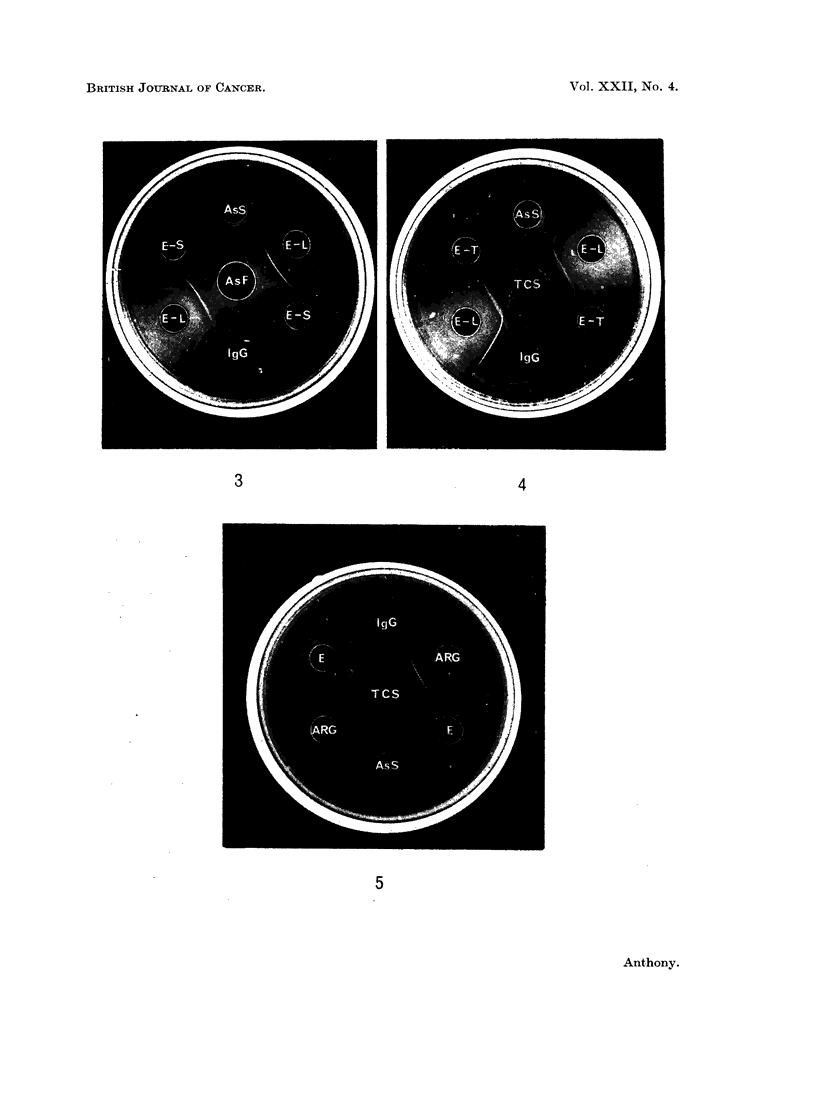

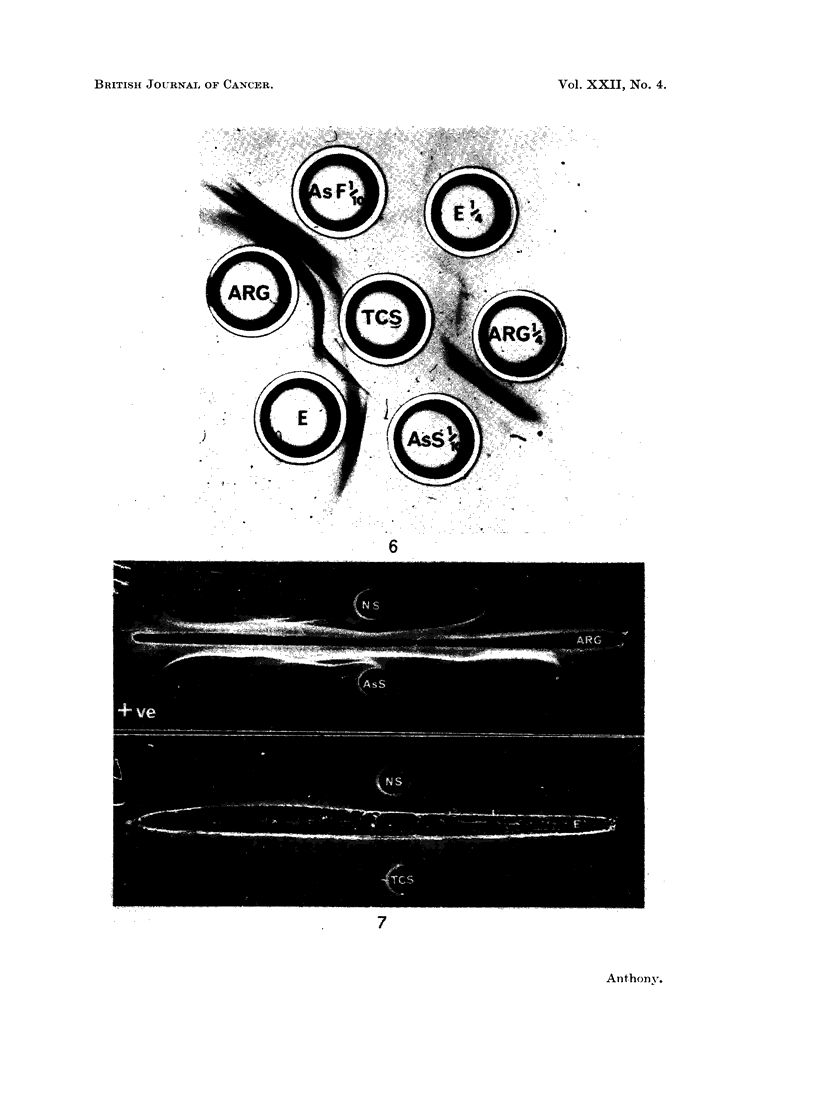

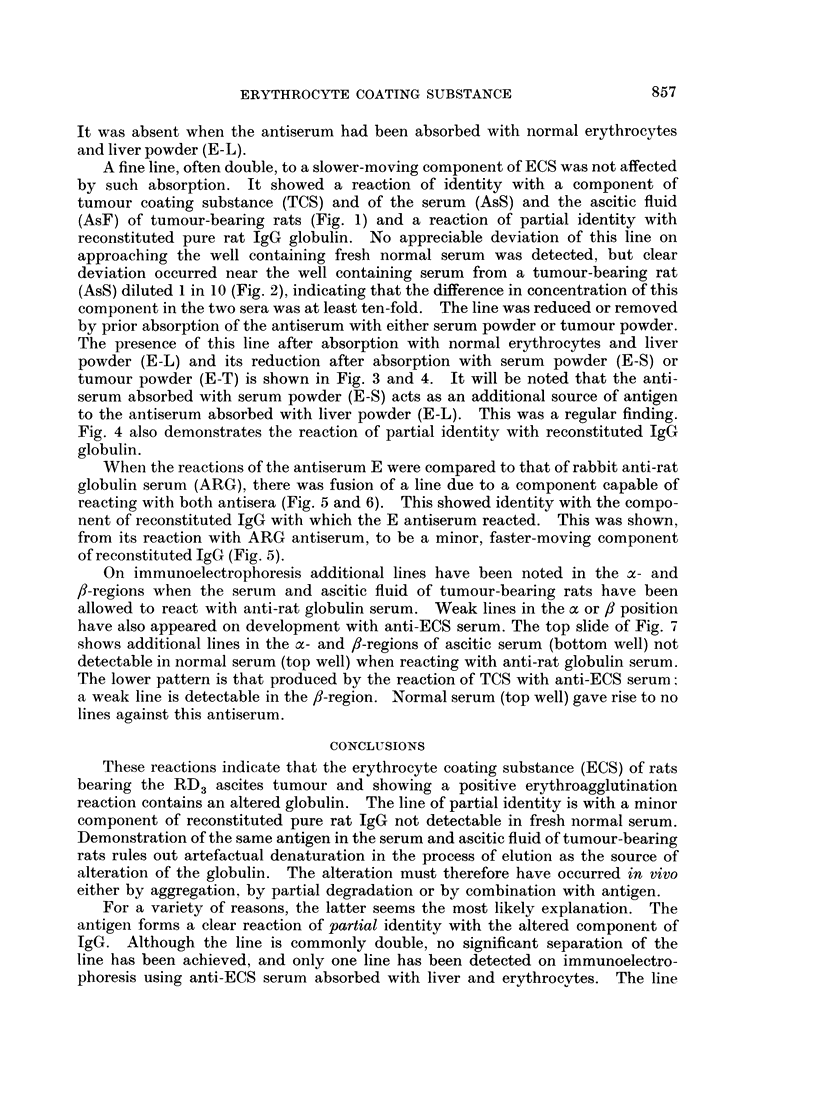

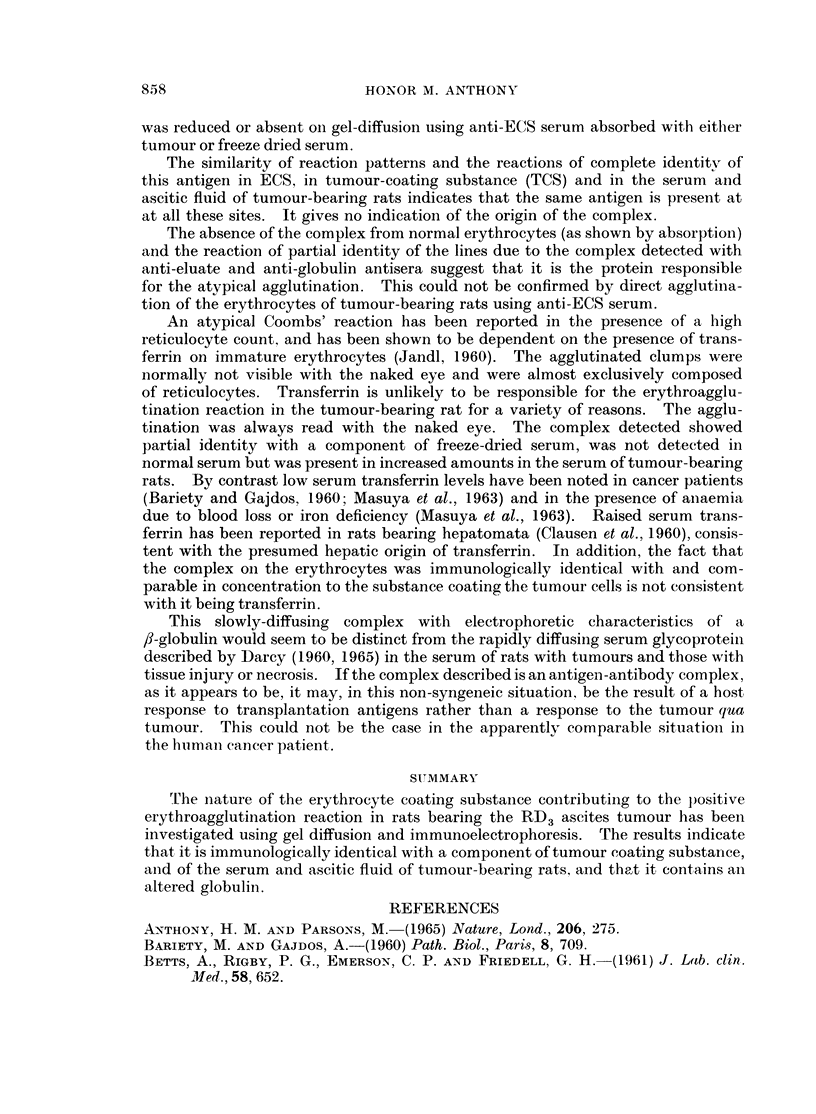

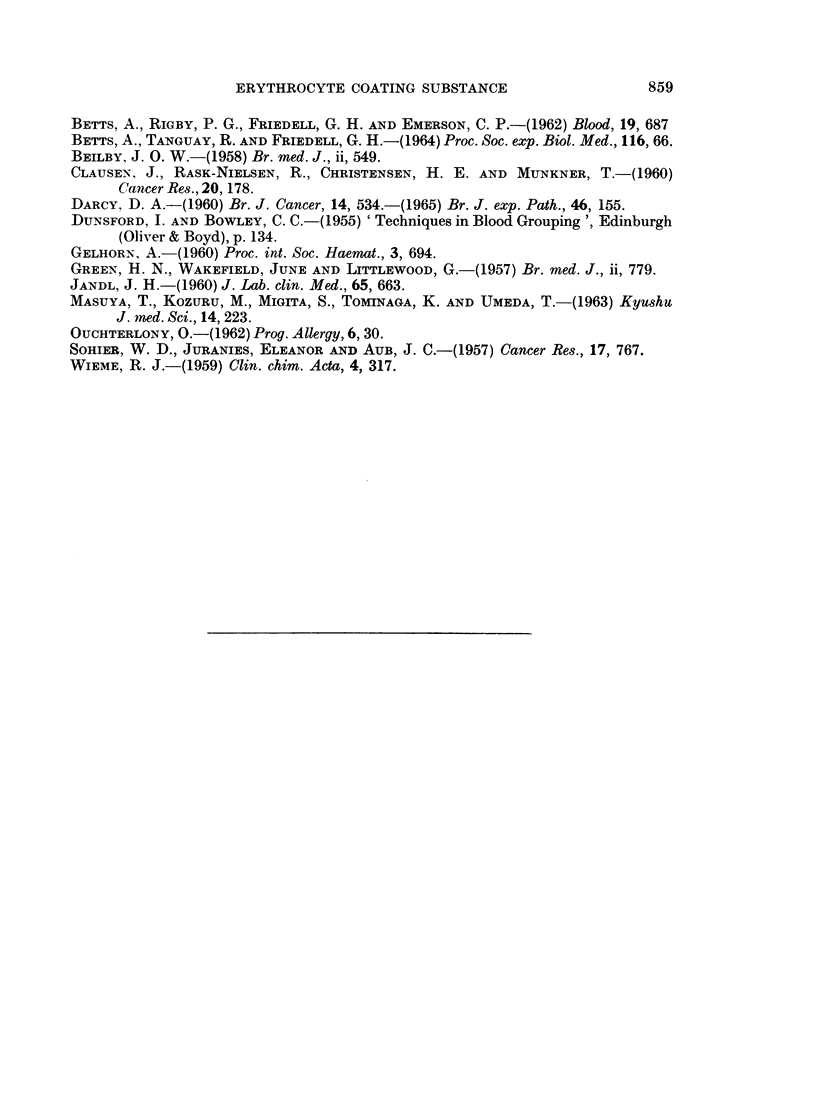


## References

[OCR_00328] Anthony H. M., Parsons M. (1965). Globulin on cells of cancer patients.. Nature.

[OCR_00329] BARIETY M., GAJDOS A. (1960). [Hyposideremia and hyposiderophilinemia in the course of cancers and especially in the course of bronchopulmonary cancers].. Pathol Biol.

[OCR_00331] BETTS A., RIGBY P. G., EMERSON C. P., FRIEDELL G. H. (1961). Experimental studies in the anemia of malignancy. I. The anti-globulin test in tumor-bearing hamsters.. J Lab Clin Med.

[OCR_00337] BETTS A., RIGBY P. G., FRIEDELL G. H., EMERSON C. P. (1962). Erythroagglutination reactions in cancer, tuberculosis and pregnancy.. Blood.

[OCR_00343] CLAUSEN J., RASK-NIELSEN R., CHRISTENSEN H. E., MUNKNER T. (1960). Two transplantable mouse hepatomas associated with an increase of metal-combining beta-globulin (transferrin) in serum.. Cancer Res.

[OCR_00347] DARCY D. A. (1960). A quantitative study of a serum protein associated with tissue growth. Values found in tumour-bearing rats.. Br J Cancer.

[OCR_00353] GREEN H. N., WAKEFIELD J., LITTLEWOOD G. (1957). The nature of cancer anaemia and its bearing on the immunological theory of cancer.. Br Med J.

[OCR_00354] JANDL J. H. (1960). The agglutination and sequestration of immature red cells.. J Lab Clin Med.

[OCR_00356] MASUYA T., KOZURU M., MIGITA S., TOMINAGA K., UMEDA T. (1963). CLINICAL AND EXPERIMENTAL STUDIES ON THE METABOLISM OF TRANSFERRIN. I. THE SERUM TRANSFERRIN LEVELS IN VARIOUS DISEASES.. Kyushu J Med Sci.

[OCR_00362] SOHIER W. D., JURANIES E., AUB J. C. (1957). Hemolytic anemia, a host response to malignancy.. Cancer Res.

[OCR_00363] WIEME R. J. (1959). An improved technique of agar-gel electrophoresis on microscope slides.. Clin Chim Acta.

